# Green and Advanced Food Freezing/Anti-Freezing Technologies: Recent Advances and Future Perspectives

**DOI:** 10.3390/foods15213922

**Published:** 2026-11-02

**Authors:** Rui Hu, You Tian

**Affiliations:** 1 School of Food Science and Engineering, South China University of Technology, Guangzhou 510641, China; hurui2023@163.com; 2 Academy of Contemporary Food Engineering, South China University of Technology, Guangzhou Higher Education Mega Center, Guangzhou 510006, China

## 1. Introduction

For nearly 150 years, industrial refrigeration and freezing have underpinned the stability of global food supply chains [1,2]. By depressing kinetic energy and arresting microbial propagation and biochemical decay, freezing effectively “pauses” the biological clock of perishable commodities. Yet, this preservation mechanism has always functioned as a double-edged sword [3]. In conventional freezing processes, the slow and unguided transition of water into ice induces the formation of coarse, irregular extracellular ice crystals [4], which mechanically shear cell walls in plant tissues [5], sever myofibrillar filaments in animal muscle [6,7], and inflict lethal cryoinjury on functional microorganisms or cell suspensions [8,9].

At the macroscale, this damage manifests as excessive post-thaw drip loss, severe textural softening, nutritional leaching, and the development of rancid or fermented off-flavors [10,11]. Concurrently, traditional cold chain systems rely heavily on energy-intensive vapor-compression refrigeration and brute-force air circulation, carrying substantial carbon footprints and high operational costs [12]. As the global food system confronts the dual pressures of climate change and heightened consumer expectations for clean-label, farm-fresh quality, the industry can no longer rely solely on legacy freezing paradigms [13]. A systemic transformation is required—one that synchronizes advanced, green physical-field freezing technologies with bio-derived, highly efficient anti-freezing interventions to control ice crystallization at its thermodynamic roots.

In recent decades, to combat these inherent thermodynamic challenges and align with global sustainability goals, researchers and the food industry have sought green, advanced, and highly efficient freezing and anti-freezing technologies [14,15]. This Special Issue addresses the critical need for an iterative upgrade of the frozen food industry by bridging traditional cryogenics with novel multi-physical field interventions, predictive artificial intelligence (AI), and bio-derived cryoprotectants. Together, these original research articles and comprehensive reviews provide a roadmap for the future of food preservation, quality assessment, and biochemical stability.

## 2. Special Issue Overview

The scientific topic of this Special Issue, titled Development of Green and Advanced Food Freezing/Anti-Freezing Technologies, aims to provide an overview of research on green and advanced freezing/anti-freezing technologies. Researchers from China, the United States of America, and Malaysia contributed their valuable works to this Special Issue. The contents broadly cover multiphase dynamics, biochemical stability, and the structural evolution of biological matrices under subzero stress, greatly expanding our academic understanding and scientific perspective on freezing and anti-freezing technologies.

### 2.1. Molecular Rules of Antifreeze Peptide Cooperativity

Yuan et al. (Contribution 1) resolved a longstanding debate in cryobiology regarding whether peptide length heterogeneity confers synergistic or antagonistic ice recrystallization inhibition (IRI). Employing atomistic molecular dynamics simulations with the TIP5P water model and the CHILL+ algorithm, the authors analyzed soybean- and collagen-derived peptides across *β*-sheet and α-helical conformations. Their findings demonstrated that mixing peptides of different chain lengths is not universally beneficial; rather, the outcome is dictated by secondary-structure compatibility. Systems pairing disparate chain lengths of *β*-sheet peptides consistently enhanced ice suppression, driven by productive intermolecular peptide–peptide hydrogen bonding and favorable surface area-to-volume reconfiguration. Conversely, mixing highly active individual α-helical peptides resulted in conformational interference, reduced intermolecular coupling, and a near-total loss of anti-freezing activity. This provides clear molecular design rules for formulating clean-label cryoprotectant cocktails from upcycled plant proteins.

### 2.2. Longitudinal Biochemical and Flavor Drift in Frozen Plant Matrices

Zhao et al. (Contribution 2) conducted an integrated 12-month evaluation of kiwifruit pulp maintained under commercial storage conditions (−20 °C). While conventional macro-indicators such as total soluble solids (TSS) and titratable acidity (TA) remained largely stable, multi-dimensional profiling revealed profound microscopic decay. Ice crystal recrystallization and cellular rupture provoked a >50% decline in carotenoids, a 29.45% degradation of total chlorophyll, and an irreversible collapse of ascorbic acid (Vc) and total phenolics, accompanied by significant malondialdehyde (MDA) accumulation from lipid peroxidation. Crucially, E-nose and HS-SPME-GC-MS volatilomics demonstrated an abrupt flavor profile inversion: beyond 9 months, fresh ester notes vanished, replaced by objectionable lipid-oxidation and fermentation volatiles such as (E)-2-octenal, cis-2-penten-1-ol, and ethanol. This study established a rigorous 9-month industrial shelf-life ceiling for premium pulp processing, demonstrating that subzero storage is an active biochemical timeline rather than a static holding state.

### 2.3. Dynamic Impact and Freezing Kinetics of Agro-Industrial Byproducts

Hu et al. (Contribution 3) expanded droplet impact cryodynamics beyond simple water by investigating porcine bile—an abundant agro-processing byproduct (exceeding 300,000 tons annually) essential for pharmaceutical bile powder production. Utilizing high-speed imaging on supercooled substrates (−10 °C~−20 °C), the authors characterized droplet dynamics through five dimensionless numbers: Weber (*We*), Reynolds (*Re*), Bond (*Bo*), Ohnesorge (*Oh*), and Prandtl (*Pr*). They demonstrated that higher impact velocities (*We* > 3) promote widespread film spreading, whereas lower substrate temperatures suppress droplet recoil by rapidly extracting latent heat and establishing a solid anchoring layer. Droplet freezing time drops by up to 45% at −20 °C, yielding flat, thin ice sheets at high We, as opposed to smooth hemispherical ice caps at *We* < 1. This work provided the exact fluid dynamic models required to design high-efficiency, energy-saving spray-freeze-drying towers for animal byproducts.

### 2.4. Glass Transition and Pectin Mechanics in Combined Drying

Li et al. (Contribution 4) addressed the energy-intensive nature of vacuum freeze-drying by evaluating a combined freeze-drying and hot air drying (FD-HAD) strategy for apple cubes. The study revealed that product shrinkage and structural deformation are governed by the dynamic interplay between the sample’s core temperature (*T_w_*) and its glass transition temperature (*T_g_*). When *T_w_* exceeds *T_g_*, the tissue transitions into an unstable rubbery state, characterized by a collapsing relaxation modulus (*E*_1_) and relaxation index (*n*) in Maxwell mechanical modeling. Mechanistically, prolonged exposure to the hot air stage accelerates the degradation of water-soluble pectin (WSP) and elevates the esterification degree of chelator-soluble pectin (CSP), weakening the middle lamella and calcium cross-linking network. The authors identified the precise transition moisture threshold (14 h pre-drying) at which structural rigidity is retained, providing a thermodynamic roadmap for low-energy fruit dehydration.

### 2.5. Intelligent and Multi-Physical Meat Processing

The review (Contribution 5) by Xia et al. systematically consolidated the evolution of meat freezing and thawing, transitioning from foundational thermodynamic principles to the integration of multi-physics methods and AI. They reviewed how physical fields—such as cavitation-induced nucleation in ultrasound-assisted freezing (UAF), electrostatic dipole orientation in electric field-assisted freezing (EFAF), and phase-diagram manipulation in high-pressure freezing (HPF)—mitigate cellular tearing and reduce thawing loss. Crucially, they identified the convergence of AI with computational fluid dynamics (CFD) and finite element modeling (FEM) as the solution to longstanding industrial bottlenecks. By coupling predictive neural networks with non-destructive spectral sensing, the authors demonstrated how real-time, closed-loop control can resolve heating non-uniformities in radiofrequency thawing and adapt process parameters dynamically to variable meat geometries.

## 3. Future Directions and Knowledge Gaps in the Frozen Food Industry

While the studies in this Special Issue present groundbreaking advancements, they also illuminate persistent knowledge gaps that must guide future inquiry.

### 3.1. Bridging the Multi-Scale Translation Chasm

There still remains a disconnect between micro-scale thermodynamics and macro-scale industrial engineering. The droplet impact dynamics detailed by Hu et al. and the atomistic hydrogen-bonding networks uncovered by Yuan et al. provide high-resolution mechanistic insights. However, translating the behavior of a single porcine bile droplet or a nano-scale peptide lattice into the turbulent, heavily loaded environment of a continuous blast-freezing tunnel remains highly complex. Future research must focus on scaling these phenomena through advanced surrogate modeling, where Computational Fluid Dynamics (CFD) and Finite Element Methods (FEM) are embedded with AI to account for the heterogeneous composition of bulk food matrices.

### 3.2. Overcoming Energy and Capital Constraints

Advanced physical fields offer undeniable quality benefits, but their industrial adoption is restricted by economic feasibility. High-pressure shift freezing (HPSF) and electric field-assisted freezing (EFAF) require specialized, capital-intensive equipment and face challenges related to electrode configuration and high-voltage safety in wet processing environments. Similarly, hybrid methods like FD-HAD demand precise, continuous monitoring to prevent the product temperature from crossing the glass transition threshold and causing structural collapse. The industry requires the development of retrofittable, low-cost physical field emitters and the standardization of protocols to make these green technologies accessible to mid-sized processors.

### 3.3. Dynamic Supply Chain Management

The longitudinal findings of Zhao et al. definitively prove that frozen storage is an active biochemical timeline, not a state of suspended animation. Relying strictly on calendar-based expiration dates for frozen commodities ignores the realities of temperature abuse during transport and the slow, insidious drift of lipid peroxidation and ice recrystallization. Future cold-chain logistics must integrate miniaturized and intelligent sensors, such as handheld NIR devices, smart E-noses and time temperature indicators (TTI), to monitor molecular volatile signatures and moisture states in real time. This dynamic tracking will allow producers to reroute or process commodities based on empirical biochemical quality rather than arbitrary dates.

## 4. Conclusions

This Special Issue fundamentally redefines our approach to low-temperature food preservation. By migrating away from passive, brute-force thermal extraction, modern freezing and anti-freezing technologies now embrace active, intelligent modulation of the water-to-ice phase transition. Through the synergistic application of multi-physical fields, the thermodynamic mapping of viscoelastic glass transitions, and the fluid dynamic modeling of complex biological byproducts, researchers are engineering processes that preserve microscopic cellular integrity while slashing energy consumption. Furthermore, longitudinal biochemical tracing proves the necessity of strict quality timelines, while atomistic simulations unlock the design rules for upcycling agricultural proteins into highly active, clean-label cryoprotectants. Ultimately, mastering the physics and biochemistry of the subzero microworld is not merely an exercise in maintaining product texture; it is a critical imperative for reducing global food waste, maximizing resource recovery, and engineering a more sustainable and equitable global food system.

## Data Availability

No new data were created or analyzed in this study. Data sharing is not applicable to this article.
